# Professionals’ health conceptions of clients with psychosocial problems: An analysis based on an empirical exploration of semi-structured interviews

**DOI:** 10.1016/j.ijnsa.2023.100120

**Published:** 2023-03-05

**Authors:** Fia van Heteren, Nadine Raaphorst, Sandra Groeneveld, Jet Bussemaker

**Affiliations:** a Department of Public Health and Primary Care/Health Campus The Hague, Leiden University Medical Centre, P.O. Box 13228, 2501 EE The Hague, The Netherlands; b Institute of Public Administration, Leiden University, Leiden, The Netherlands

**Keywords:** Concept formation, Culture, Health, Health personnel, Interview, Professionalism, Methods, Qualitative methods, Social workers

## Abstract

**Background:**

The care of clients with complex psychosocial problems involves diverse frontline professionals such as general practitioners, psychiatric nurses, police officers, social support consultants and debt counselors. As these professionals have different professional backgrounds and work in different organizations, their health conceptions, or beliefs about what constitutes health and how this should be pursued, may also differ. Having an understanding of various frontline professionals’ health conceptions is relevant, as these may affect interprofessional collaboration in their work with clients with psychosocial problems.

**Objective:**

To understand various frontline professionals’ health conceptions.

**Design:**

Inductive qualitative approach.

**Setting:**

The Hague, the Netherlands.

**Participants:**

Various frontline professionals from social welfare, general healthcare and mental healthcare, working with clients with complex psychosocial problems.

**Methods:**

Between September 2020 and April 2021, 23 in-depth semi- structured interviews were conducted with frontline professionals in social welfare, general healthcare and mental healthcare. Based on these interviews, this paper analyzes frontline professionals’ health conceptions. After transcription, all interviews were imported into ATLAS.ti for analysis. An iterative process of thematic analysis was used to identify health conception dimensions.

**Results:**

The paper found that frontline professionals’ health conceptions differ in three main aspects: 1) health definitions, 2) alignment with clients and 3) contextualization of clients’ health.

**Conclusions:**

The main implication of this research is that this inductive analysis of health conceptions provides a first building block in theorizing frontline professionals’ health promotion practices.

**Tweetable abstract:**

Knowing about professional's health conceptions gives insight into how health is understood and how good health can best be achieved, which is important in caring for vulnerable clients.

## Contribution of the paper

### What is already known about the topic

• Studies on the health conceptions of professionals focus on physicians, while the health conceptions of the diverse professionals involved in care may affect health-promoting practices, collaborative practices, and in turn, patient outcomes.
• Professionals’ health conceptions may consist of various dimensions.

### What this paper adds

• This research demonstrates that various frontline professionals’ health conceptions not only consist of health definitions, but also include beliefs about what should be expected of clients and other stakeholders involved in becoming healthy.
• This research found that health conceptions consist of three dimensions: health definitions, aligning with clients and contextualization of the health of clients, each with its own interpretations.
• Professionals stress that they are not always able to act upon thier ideal health conceptions because of limited resources and complex health conditions related to clients. This research suggests that ffurther research should focus on the actual health promotion practices of professionals.

## 1 Introduction

Combinations of physical, social and psychological issues are referred to as psychosocial problems (Van Hook, 2004). People with such complex problems may, for example, have debts, and suffer from depression or stress, as well as chronic headache. The care for people with multiple, complex and chronic conditions is becoming a major burden for frontline professionals from various domains (Grumbach and Bodenheimer, 2004). Clients with psychosocial problems therefore often receive care from many different professional disciplines, such as general practitioners, social-psychiatric nurses or social workers, who are to some extent working together across professions and organizations. Broad conceptions of health, referring to beliefs ‘that guide health professionals in their attempts to understand, explain, make sense of, and respond to health-related phenomena’ (Levesque and Li, 2014, p. 629: 629), are considered valuable in this context, since they have the potential to bridge gaps between medical healthcare, mental healthcare and social welfare, thereby possibly de-medicalizing societal problems (2011; M. Huber et al., 2016).

There is a lack of insight into the health conceptions held by frontline professionals in general healthcare, mental healthcare and social welfare (Armstrong and Swartzman, 1999; Colombo et al., 2003; Levesque and Li, 2014). Understanding professionals’ health conceptions is relevant, as these may affect health promotion and interprofessional collaboration. Our study therefore develops a conceptualization of health conceptions grounded in the definitions expressed by professionals relating to health and the beliefs about required practices. The central research question is as follows: How can the health conceptions of frontline professionals in (mental) healthcare and social welfare be conceptualized?^1^ To answer this question, we conducted a qualitative interview study among diverse frontline professionals in social welfare and general- and mental healthcare. These professionals all work with clients with psychosocial problems, and collaborate across professional and organizational boundaries.

This study contributes to existing scholarship in three ways. First, the literature on health conceptions mainly focuses on cultural differences in patient groups, and on how conceptual differences between health professionals and patients impede therapeutic processes (Armstrong and Swartzman, 1999; Levesque and Li, 2014; Pachter, 1994). We contribute by studying health conceptions held by professionals rather than lay health conceptions. Second, studies focusing on the health conceptions of health professionals (Huber et al., 2011) focus on physicians, while the health conceptions of diverse professionals involved may affect health-promoting practices, collaborative processes, and, in turn, patient outcomes. Involving other types of professionals than physicians in the study of health conceptions is relevant in order to grasp a variety of insights. Third, the literature suggests that health conceptions are broader than health definitions and that they consist of various dimensions. We will contribute by further conceptualizing these dimensions. In order to better understand how these frontline professionals work as an integrated body, it is necessary to understand their health conceptions.

In what follows, we first conceptualize health conceptions by drawing on health care literature. Our research context and mainly inductive methodology are then explained, followed by a presentation of our findings working towards a grounded conceptualization of professional health conceptions. We conclude with a discussion and avenues for future research.

## 2 Literature review: defining health conceptions

Notwithstanding some medical studies about what health conceptions include, such as the ability to achieve or exercise a cluster of basic human activities (Venkatapuram, 2013), only few social scientists clearly define what they mean by health conceptions. Levesque and Li (2014) refer to health conceptions as explanatory models ‘of health and illness, which include beliefs about possible causes of illness, onset and evolution of symptoms, pathophysiology of illness, severity of illness, and possible treatments’ (Kleinman, 1978). This definition primarily focuses on beliefs about illness, rather than health. While illness and health are related, existing research on how people define *health* has pointed out that it could be seen as more than the absence and, hence, treatment of disease (Hjelm, 2006). In addition, health conceptions may be classified not only by beliefs about what health is, but also by what should be done to sustain and improve health and how those involved should behave towards each other (Colombo et al., 2003). For this reason, we define health conceptions as beliefs about what health is, about the factors that affect people's health and about those practices that promote health (Colombo et al., 2003; Levesque and Li, 2014). Professionals’ health conceptions thus include definitions of what constitutes health, beliefs about required practices of clients and their environment, and beliefs about how the professionals involved should behave towards clients, their surroundings and each other.

Health conceptions are dynamic (Bircher, 2005; Levesque and Li, 2014), which means that they not only shape how new knowledge and experiences are interpreted, but are also shaped *by* new knowledge and experiences. This implies that health conceptions serve as a frame through which experiences are interpreted and explained (Goins et al., 2011; Kleinman, 1978; Levesque and Li, 2014; Torsch and MA, 2000). In our study this means that professionals’ health conceptions affect, for instance, the information they pick up, deem important and act upon in interactions with clients. New information and knowledge are assimilated ‘to fit into existing cognitive structures or schemas’ (Levesque and Li, 2014) and beliefs about health could be adapted by new experiences.

Research about health conceptions mainly focuses on clients’ lay perspectives of health, and explores differences in health conceptions based on demographic factors (Barnes et al., 2008; Dubbin et al., 2013; Robertson, 2006). Recently, however, there has been growing awareness of the importance of various stakeholders’ health conceptions, including professionals. For instance, Huber and colleagues (Huber et al., 2011, 2016) evaluated among stakeholders such as health care professionals, patients, policy makers and insurers, the support for their conceptualization of health as positive health: ‘health as the ability to adapt and to self-manage in the face of physical, social and emotional challenges’ (Huber et al., 2011). Positive health includes six dimensions: bodily functions, mental functions and perception, spiritual/existential dimension, quality of life, social and societal participation, and daily functioning. They found significant differences between groups, with patients valuing all dimensions equally, while physicians mainly assessed health biomedically (Huber et al., 2016). However, as frontline professionals in social welfare and mental healthcare were not included in this study, what is missing is clarity about how the broader range of professionals providing care define and pursue health in caring for clients with complex problems.

Following our definition of health conception and Huber and colleagues (Huber et al., 2016), health conceptions consist of different dimensions. To be able to develop an empirically grounded conceptualization of health conceptions requires an inductive and explorative study including professionals from various professional backgrounds.

## 3 Research approach

The study takes an inductive qualitative approach (Nowell and Albrecht, 2019). The inductive logic fits with the focus on theory building, as empirical exploration is needed to be able to form a conceptualization. The design is fitting because the health conceptions of professionals are highly understudied. The data stem from a qualitative study focused on diverse frontline professionals working with clients with combined psychosocial problems in the Netherlands. The study focuses on the perceptions and interpretations of people themselves, which is important to research because professionals’ perceptions are suggested to play a leading role in professionals’ practices (Levesque and Li, 2014).

### 3.1 Research setting

The study was carried out with frontline professionals working in social welfare and (mental) healthcare in The Hague, which is a large city in the west of the Netherlands with a population of approximately 500.000. In some areas of this city, the number of years lived in good health is among the lowest in the Netherlands. Psychosocial problems are common, which is reflected in the occurrence of severe psychiatric conditions, confused behavior, a high risk of anxiety disorders and depression and the increase of dementia. Such problems are particularly common among low-income residents and are often found in combination with social and medical problems (Haaglanden, 2021). To deal with these problems, the city implements policy initiatives that require high levels of collaboration between various professionals and organizations. Hence, because this research needs a diverse sample of professionals to construct a conceptualization, psychosocial care in the Hague is a context that is well suited for this purpose.

### 3.2 Methods and data

Respondents were selected on theoretical grounds. All 23 respondents are frontline care professionals in social welfare, mental or general healthcare working with clients with psychosocial problems in The Hague. This exploratory study aimed to gather a multitude of perspectives and experiences to be able to conceptualize, because different professionals may think differently about health. The sample therefore consisted of various kinds of frontline professionals, such as: out-patient attendants, psychiatric nurses, community police officers and general practitioners. Frontline care professionals from different organizations and different neighborhoods in The Hague were interviewed. All respondents have been doing frontline work for years (respondent characteristics in Appendix A). Four respondents are professionals in general medicine, six are professionals in mental health and thirteen respondents are professionals in social welfare. The first author recruited the first respondents through contact with a gatekeeper in a network organization in the field of mental healthcare. Following this introduction to the field, the author used snowball sampling, based on referrals from initially sampled respondents, to recruit respondents with the above criteria in mind. The advantage of this sampling strategy is that it allows the researcher to reach populations that are otherwise difficult to sample (Johnson, 2014). Moreover, this respondent selection made it possible to study health conceptions in a frontline care context where professionals are all caring for clients with complex problems and where there is a major focus on interprofessional collaboration.

We used semi-structured interviews (see Appendix B) to gain insight into respondents’ health conceptions. Interviews give insight into people's perceptions and the meanings they attach to situations (e.g. Maynard-Moody et al., 2003). Our definition of health conception served as a sensitizing concept in constructing the interview questions. The concept is sensitizing rather than definitive, because it lacks a specification of attributes or benchmarks which would allow a clean-cut identification of a specific instance of its content. Instead, it gives a general sense of reference and guidance in approaching empirical instances (Blumer, 1954). Following Blumer (1954), the use of sensitizing concepts matches the inductive nature of this study, recognizing that what we are referring to by any given concept inspired by the existing literature may shape up in different ways in each empirical instance. As such, open questions were asked about health views, pursuing health, why it is important, how they interact with other involved stakeholders. Respondents were also asked to give examples of daily work activities and to reflect on their experiences at work. The interviews, varying between 45-90 min in duration, were conducted between September 2020 and April 2021. Most interviews took place at a location chosen by the respondents, although some interviews were held online because of Covid-19 restrictions. They were all audio-tape recorded, transcribed verbatim and imported into ATLAS.ti for analysis. Informed consent was given by all respondents. This study was registered and approved by the Medical Ethical Review Committee of Leiden, The Hague and Delft (N20.158).

The analytical process moved back and forth between transcripts, analytical memos, emerging themes, and theory. We used thematic analysis to identify key themes relevant to our research question, and followed different steps in our analysis outlined by Braun and Clarke (2006).The iterative and recursive analysis began with initial open coding, during which categories were assigned to the themes in the transcripts. We coded the content of the full transcripts in detail but paid special attention to codes that were relevant to our sensitizing concept of ‘health conception’. We then searched for more abstract themes by comparing the codes within and between the transcripts and by organizing them into codes and more general code families (Braun and Clarke, 2006). These code families were subsequently refined by going back to the coded transcripts and rereading them to check whether the themes reflected the meanings found in them. After multiple rounds of coding, the general code families reflect our final health conception dimensions (coding table in Appendix C). As our aim was to construct a conceptualization, we paid particular attention to the internal homogeneity and external heterogeneity of the dimensions (Braun and Clarke, 2006). This holds that data ‘within themes should cohere together meaningfully, while there should be clear and identifiable distinctions between themes’ (Braun and Clarke, 2006). In the coding process, conflicts in the emerging patterns were explicitly looked for, which led to the accounts and interpretations being re-examined. Lastly, in the discussion stage, we looked back at the sensitizing concept and theoretical framework to reflect on how our interpretations differ from the existing literature.

To increase the validity and reliability of our research, we followed several procedures outlined by Krefting (1991): negative case analysis, keeping notes about our data collection and analysis and peer examination. Throughout the analytical process, we searched the data for insights that would disprove our emerging insights, we wrote memos and kept logs on the steps taken in the coding process and multiple researchers were involved in interpreting the findings.

For the purposes of presentation, quotes were translated into English with the intention of maintaining the original meaning. The quotes that were used in the analysis serve an illustrative purpose, best representing the themes found. Information that could identify respondents is omitted from the findings. The remainder of this article presents and discusses the main themes and their interpretations.

## 4 Frontline professionals’ health conceptions: a three-dimensional conceptualization

This section presents an analysis of health conceptions held by frontline professionals in social welfare and general- and mental healthcare. Based on the inductive analysis of the interview data, we found that health conceptions consist of three dimensions: (1) health definitions, (2) alignment with clients and (3) contextualization of health (see Table 1). In what follows, the different health conception dimensions and their substantiations will be presented. Following examples from the data, we will conceptualize further by describing each dimension and its boundaries in more general terms. In the second part of the results section, we will address the interplay between the different health conception dimensions in our empirical data.

**Table 1:** Health conceptions: dimensions.

Dimensions	Substantiations
**Health definitions**	The ways and extent to which a client is seen as healthy.
*Competence and behavior*	Health as the extent to which people can rely on themselves or their network to do what they want by being strong, vital and communicative.
*Mental health*	Health as the extent to which a person is mentally healthy and experiences stability.
*Physical health*	Health as the extent to which a person is without physical complaints and without threat to life.
**Alignment with clients**	The ways and extent to which horizontal and vertical distances in the professional-client relationship are kept to a minimum.
*Being approachable*	The extent to which the professional is close to a client's lived experience.
*Seeking alignment*	The extent to which and the ways in which the professional invites the client to share their preferences and takes these into account in the treatment plan.
**Contextualization of problems**	The extent to which and the sources used by professionals to place clients in their broader context to understand what actions will be appropriate in helping them.
*Assessing social context*	The extent to which and how a client's social circle is assessed and connected with.
*Assessing other problems*	The extent to which and how problems emerging in other life areas are assessed.

### 4.1 Dimension A: defining health

This first health conception dimension refers to the ways in which and the extent to which a client is seen as healthy. By defining health, frontline professionals equip themselves with ideas about when a client could be considered healthy and thus what they, both professional and client, should see as goals to work towards. Frontline professionals have varying ideas about whether certain aspects of health definitions should be considered as goals when working towards healthy clients. We found three distinct ways in which health definitions are substantiated by professionals, namely: competence and behavior, mental health and physical health, which will be discussed below.

#### 4.1.1 Competence and behavior

Health as competence and behavior encompasses the extent to which a client can rely on themselves and their network to live as they wish. Accounts about competence and behavior are mostly about clients showing that they can live independently by using skills and knowledge such as being powerful, vital or health literate. Health literacy could help clients to make substantiated decisions about their health. In this view, healthy clients are thus knowledgeable and independent, and can communicate, self-manage and self-learn, they deal with problems, participate in society, and recognize their patterns and signals (of stress). The following examples show the importance of competence and behavior as a health definition for frontline professionals working with clients with combined problems:

*‘[…] [along with] all sorts of problems that someone […] [may have,] […]the coping capacity is the extent to which someone can, from themselves, have the power and tools to deal with that. (respondent 1, social worker)’*

*And*

*‘[…] this one man […] is much healthier in a way […], because he makes sure his house is tidy and that is some kind of resilience that the other kid doesn't have yet. [..] He still uses cocaine, but he has more control […] and he doesn't have friends visiting him at 2 a.m.; […] He remains below the police radar and doesn't create a nuisance (respondent 3, police officer).’*

This definition of health as competence and behavior is a particular one. The examples show that a client is considered healthy when they are competent to deal with problems themselves or are able to ask others in their social circle for help. The examples show that health is not defined in a physical or mental way, but rather as the skill of being in control of your own and your social environment's behavior. As such, healthy clients have the individual potential to do well in society despite the many problems they may have, because they are able to take control, set boundaries and know when and how to ask for help.

#### 4.1.2 Mental health

Mental health, the second health definition, can be distinguished from competence and behavior. Whereas frontline professionals with a competence and behavior health definition see clients as healthy when they show great individual potential, professionals with a mental health definition stress that clients are healthy when they feel healthy in their own experience. As the following examples show, clients are seen as healthy when they can live life the way they prefer, without necessarily showing great potential or competent behavior. When clients have an illness, but they do not experience this as problematic in their lives, they can still be healthy in this health definition. This definition stresses that health is very personal and it is related to the client's perspective of life. The following examples show that respondents use different words to describe mental health, but their interpretations are similar.

*‘This [,health,] is very personal. [Clients are healthy] When [they] […] have no complaints and don't experience barriers to being in balance’ (respondent 4, social case manager).’*

And

*‘The client perception is important, because they feel healthy when they experience few obstacles in daily life. […] What we see helps here is to have a goal in life, for example through a job and daily structure. This kind of perspective could help clients not to fall into a depressive spiral (respondent 5, client supporter).’*

These examples show that mental health is personal and perceptive, which is argued by most respondents. However, some respondents make an exception when actors in the clients’ surroundings experience the client to be unhealthy, for example through danger to themselves or others. Therefore, in their words, clients are mentally healthy when they ‘*don't stand out*’ in society (respondent 22, police officer)’.

#### 4.1.3 Physical health

Physical health is the third kind of health definition in our data, which does not focus on client behavior or experience, but instead on a client's physical abilities, like being able to move your body, having a healthy weight or being without any serious physical illness. The following two quotes illustrate this conceptualization of a physical health definition.

*’Clients are healthy when they are not in a life-threatening situation (respondent 20).’*

And

*‘Objectively, someone with no physical complaints is healthier than someone who chronically needs a wheelchair.’ (respondent 5).*

These respondents, thus, define health as having good physical abilities. Nevertheless, even though they strive for good physical health, being perfectly healthy physically does not seem possible in the eyes of these respondents who work with clients with multiple problems.

Health definitions are particular in the sense that they reflect ideas about what goals professionals, clients and their environment should work towards. We have seen that these goals could relate to competence and behavior, mental health and physical health.

### 4.2 Dimension B: alignment with clients

Alignment with clients, the second health conception dimension, can be distinguished from defining health. Whereas health definitions help respondents to make sense of which goals they could work towards, health definitions do not necessarily imply how they perceive that these goals should be achieved. As the following section shows, health conceptions are constructed not only by health definitions, but also by perceptions about the ways in which - and the extent to which - horizontal and vertical distances in the professional-client relationship should be minimized. We conceptualize alignment as professionals’ intention to come close to a client's lifeworld, experiences and preferences by minimizing horizontal and vertical distances. Two sorts of alignment in the professional-client relationship are at play in frontline professionals’ work with clients with psychosocial problems: seeking approachability and seeking alignment.

#### 4.2.1 Being approachable

Approachability relates to the intent to connect with clients in a relationship of trust. This happens in different ways: first, the adjustment of speech and conversation are considered important connective aspects to build the trust needed to help a client. Second, when professionals experience the lifeworld of their clients as too distant, consequently prohibiting them from helping these clients, respondents try to be approachable by being physically present in the clients’ living environment. The following quote is an example of the latter. The respondent in this example sets up an office close to their clients with the intention of being approachable to them physically:

*‘Once a week we […] had this consultation office at the local housing association […] in the neighborhood. […] This was a consultation hour for the whole neighborhood […]. […] We [wanted] to do it here […], in the neighborhood. And then they [, the youth,] came and did homework assistance […] and that is that kind of steering, helping, advising, supporting and talking. Talking until you can hardly utter another word.*^2^*’ (respondent 3, police officer).*

The example shows that being approachable physically could open up the possibility to connect further through conversation and thereafter to potentially achieve health-related goals. Approachability is especially relevant to respondents when they are caring for clients who may normally not seek help easily.

The other quotes regarding aligning similarly show how respondents create and have a relationship of trust with clients that helps them to work towards health goals together.

#### 4.2.2 Seeking alignment

Seeking alignment elicits a second kind of alignment with clients, which entails the extent to which and the ways in which a professional intends to invite clients to share their preferences and to take these into account when constructing the treatment plan. In the following quote, the frontline care professional explains how she tries to get clients to open up in order to gain an understanding of what clients find important.

‘*Questioning, probing, investigating: sometimes it's also [the case] that they don't know, you know, ignorance*.’ (respondent 11, mental health worker).

This respondent asks questions to invite clients to share their preferences. Professionals take these preferences into account to a certain extent in constructing the treatment plan. Alignment about health definitions is important to respondents because when the client agrees with or brings up the health problem, they are found to be more eager to work towards health goals. However, respondents also argue that sometimes clients have to be educated first, before they should say what they find important. This is the case, for example, when respondents think that clients are not aware of what is good for them. Thus, even though professionals try to seek alignment by opening up towards clients’ ideas about health and care, they sometimes decide to work with their own health definitions.

### 4.3 Dimension C: contextualization of problems

The data shows how, in the perception of respondents, alignment helps professionals to minimize horizontal and vertical distances to their clients, which may help to work towards health goals. However, alignment does not help professionals to gain insight into how clients’ broader context may impact their health or their opportunities to become healthy. As the following section shows, to be able to reach health goals, respondents also need to assess various contextual sources - beyond the client themselves - that help care professionals to understand what clients may be able to handle regarding being and becoming healthy, or which actions are appropriate when helping them. Professionals’ examples about contextualization of clients’ health are clustered around the two aspects: ‘assessing social context’ and ‘assessing other problems’, which will be discussed below.

#### 4.3.1 Assessing social context

Respondents often explain that they need to assess clients’ social context to decide what clients, together with their environment, could handle in becoming healthy. As such, frontline care professionals assess personal social circumstances that may impact clients’ preferences or may facilitate or hinder clients’ potential health. When assessing the personal social context, frontline care professionals try to get more information from clients about their social context, but also from their environment including family members, friends, or other professionals involved with the client. Professionals may gather such information with the client through house visits or conversations as well as by contacting other stakeholders directly. Respondents’ examples show that such contextual information could help professionals to figure out whether a client has a strong enough environment to reach self-efficacy or other health goals. The following examples show why frontline care professionals assess the client environment to figure out what health problems they could work towards.

*‘You also learn about the system surrounding them: how the children are, the partner, but also the bigger family. […] And the funny thing is that you recognize patterns. Who gets ill and how they deal with certain problems […]. You are a family doctor, and yes that has great added value.’ (respondent 16, general practitioner).*

And

*‘And the environment, I think that they also play a big role in health. Yes, I'm in an environment where people think this is important […], but [clients in] our target group […] are of course often alone, they live alone, have a small social network or none at all. Yes, if you have been an addict for years, than I think you are […] often kicked out by everybody […], you lie and you cheat. Yes, people with psychiatric problems, […] they are labeled as crazy by their environment.’ (respondent 11, mental health worker)*

The first example shows how knowing about the client environment can help clarify a client's possible health problems. The second example shows how knowing about the client environment can also increase the knowledge about possible solutions which may be appropriate for a client, including which stakeholders, professionals or relatives may be involved in these solutions. In assessing the client's social context, care professionals try to gather as much information as possible, because the care question often does not come from the client themselves, but from their social network and other professionals involved. Importantly, according to our respondents, involving other stakeholders could increase the possibility for more chronic care instead of constantly falling behind (respondent 16). In other words, clients can be helped faster and better. Even though involving the client's social context is argued to be important among all professions, respondents feel they do not always have the time and resources for this intensive work.

#### 4.3.2 Assessing other problems

Apart from assessing clients’ social context in trying to gain an understanding of their support system, frontline care professionals sometimes also assess other problems than the ones presented by the client to work out what health goals they might be able to work towards. Professionals often do this in case of multiple or vague problems that often repeat themselves. The following example shows how professionals assess other problems.

‘*To assess a client's health and needs, you need to do good research, because when you have good diagnostics, you can give good treatment. Often, professionals only treat one aspect, like depression. And if you are placed out of your house or have financial or relationship problems, to make a qualitative diagnosis you have to look into someone's broader health. So, what I think is important is the mental, but also the physical health and the different life areas, like work, living, relationships and friends, these are often much more important. […] All these kinds of things, to map these out* (respondent 11, mental health worker).

This example shows that the respondent thinks it is important to look at multiple and underlying problems when thinking about the health of clients. All respondents believe that it is important to look at health broadly, because they argue that different aspects of health and different problems influence one another. Some put it more strongly, by saying that addressing only one health aspect is not useful. Even though they believe this to be important, some respondents say they do not have the time, resources or professional role to look beyond the health area they are specialized in.

## 5 Interplay between dimensions of health conception

Based on our analysis, we have argued that frontline care professionals’ health conceptions consist of three dimensions, including health definitions, alignment with clients and contextualization of clients’ health. In the section above, we have conceptualized these dimensions. However, the analysis shows that empirically these dimensions and their interpretations often appear in combination and consequently together form the health conceptions of professionals. The number of respondents in which the dimensions co-occur is presented in a co-occurrence table, which we produced in ATLAS.ti (Table 5, Appendix D).

First, in our analysis, we noted that the different health definitions appear in combination with each other. Ten respondents consider mental health important together with health as competence and behavior. This co-occurrence may be due to the characteristics of the context in which frontline care professionals work with clients with multiple problems. More specifically, when a client is mentally healthy, they feel stable, which may coincide with behaving in ways that are seen as competent. Similarly, when one is not able to be healthy in a competence and behavior sense, this may also cause suffering in terms of mental instability. Even though the two definitions seem often closely related in practice, they are different conceptually. Mental health is about health experience, and competence and behavior are about abilities and actions. A combination of all three health definitions is rarely present in our empirical data. This happens for example, when a client has severe physical problems and cannot walk, they may not be able to live the life that they want due to little social support. Clients may also experience this suffering differently mentally. At the same time, physical problems are often triggered by mental- and social problems, such as financial difficulties.

Second, we noted that five respondents who focus on seeking alignment, also find mental health definitions important. This may be because in the mental health definition, client preferences are argued to be relevant. Similarly, when seeking alignment, respondents actively try to find out what the client preferences are by asking them questions. Conceptually, seeking alignment differs from the mental health definition as seeking alignment is a more active dimension that respondents employ to find out what the client's preferences are. Moreover, four respondents think seeking alignment and health as competence and behavior are important. This mostly happens when professionals argue that clients should be capable of personally communicating their health needs, and professionals see a less active aligning role for themselves. A possible risk here is that not every client is able to communicate their own needs, and that consequently their problems are not seen and treated. At the same time, when client perceptions are abnormal and may be dangerous, or when it is difficult to determine whether clients know that the body and mind are connected, professionals often focus less on alignment, but instead focus on what they think is important regarding their client's health.

Third, for eight respondents, seeking alignment co-occurs with contextualization of problems and for seven respondents with contextualization of social context. When a professional seeks alignment, they try to figure out the client's health perspective. In a similar line of thought, when a professional contextualizes, they try to find out what in the client context or which problems make it possible or impossible to work towards being healthy.

Fourth, the analysis shows that five respondents who define health as competence and behavior find assessing social context is important. This could be because respondents assessing clients’ social context may realize that not all clients have a social context that enables them to be healthy in the sense of competence and behavior. For example, clients without a family may be unhealthy not because they do not want to take care of themselves, but because they are not able to do this themselves or with the help of a social network. Moreover, assessing other problems co-occurs four times with mental health definitions, because when professionals treat mental health, they focus on health experience. They do not only focus on one problem that clearly presents itself, but may dare to look broader to unravel other problems clients experience.

The analysis not only shows how frontline care professionals aim to combine health conception dimensions in practice by caring for clients with combined psychosocial problems, it also shows that frontline care professionals may draw on more than one health definition at a time. This makes it challenging to disentangle health conception dimensions in frontline work. However, the conceptual differences should be taken into account when studying frontline care professionals. In the following section, we elaborate on the distinctiveness of the three-dimensional health conception and discuss how it relates to the existing literature. We also present opportunities for studying health conceptions in frontline care further.

## 6 Discussion

The health conception literature has been silent on the health conceptions of professionals. In this article, we have addressed this lacuna by further conceptualizing the health conceptions held by professionals (Colombo et al., 2003; Huber et al., 2011, Huber et al., 2016; Levesque and Li, 2014) by exploring the health conceptions of frontline care professionals. Health conceptions refer to beliefs about health and the aspects that may help promote clients’ health (Levesque and Li, 2014). In line with the health conception literature (on clients, medical professionals and other stakeholders), we argue that health conceptions consist of different dimensions (Armstrong and Swartzman, 1999; Huber et al., 2011; Levesque and Li, 2014; Pachter, 1994).

What we learn from this research is that in empirical practices, health conception dimensions and their substantiations are often connected. In line with Levesque and Li (2014), this means that professionals’ health conceptions affect, for instance, the information they pick up, consider important and act upon in interactions with clients and that beliefs about health could be adapted by new experiences, such as when providing care.

These findings underline the importance of the competence and behavior health definitions and consequently the individual responsibility of clients, which run through the analysis as an apparent theme for many respondents. Professionals assume that clients should be able to express preferences themselves, to know what they want or to take care of themselves. This assumption is even held by most professionals who focus on assessment of the client's social context. Even though an assessment of social context may reveal clients’ restrictive circumstances, some respondents argue that clients are still responsible for their own health. This focus on personal responsibility is not surprising in light of public views including the neoliberal idea that ill health is primarily self- inflicted and is dependent on an individual's unhealthy behaviors, which are considered a matter of choice (Berg et al., 2021; Galvin, 2002; Hughner and Kleine, 2004). As these views are also present in the broader care sector in which our respondents work, they may play a role in their work through socialization in both professional and organizational settings (Moyson et al., 2018; Weis and Schank, 2002; Zarshenas et al., 2014).

This research makes a threefold contribution to the health conception literature. First, as the health conception literature suggests that health conceptions may consist of various dimensions, we explored dimensions of health conceptions by inductively investigating frontline professionals working with clients with combined problems. While positive health describes six dimensions of health, these do not do justice to the dimensions we conceptualized, which relate not only to beliefs about health, but also to what is expected of clients and other involved stakeholders. The inductive element was therefore useful in our analysis to further explore health conception dimensions and it may consequently enrich the literature on health conceptions. Second, this study is one of the first to examine health conceptions of professionals by involving frontline care professionals from various professional backgrounds, who are involved in caring for clients with combined problems. Involving a diverse sample of respondents was necessary to construct a conceptualization. This conceptualization can be further validated by researchers studying larger samples of frontline care professionals. They could find, for example, how the three dimensions are connected and combined in the actual work practices and behaviors of professionals and thus, what their health conceptions look like. This is especially relevant as health conceptions shape and are shaped by new knowledge and experiences (Levesque and Li, 2014 and others). Third, although health conceptions give insight into ideal practices, professionals stress that they are not always able to act upon them because of limited resources and complex health situations of clients. More research is needed into how health conceptions manifest implicitly in actual health-promoting practices and through which mechanisms. In working with complex problems, health promotion practices may differ along professional and organizational lines, but may also relate to the tendency to work in an integrated group or to the social backgrounds of the clients with whom the professionals work (Baumann, 1961; George, 2017). This raises questions about how socialization processes may impact these frontline professionals’ actual practices, how frontline professionals are able to collaborate across borders and how they decide to promote the health of clients with varying social backgrounds. Such research is especially relevant in this context in which health promotion requires that professionals understand each other and their clients. Our analysis of health conceptions thus provides a framework to study and nuance health conceptions and their use in care practice.

Furthering the study of professional health conceptions is relevant for the scientific literature, but also for discussions among frontline professionals, managers and policymakers on how health is understood and how good health can best be achieved. Knowing about and comparing professionals’ health conceptions is important because professionals care for vulnerable clients together and misunderstanding may impact their abilities to do this. Therefore, we recommend that managers of care professionals engage in dialog about health conceptions and their use among professionals. Future research should therefore try to understand how frontline care professionals use health conception dimensions in practice. Moreover, further conceptualization would make it possible for frontline professionals and their managers to reflect on their own work through the use of these dimensions.

## 7 Limitations

This research has limitations which need reflection. A limitation of this study is that it used only the method of semi-structured interviewing. Whereas the rich narratives yield insight into the health conception dimensions perceived as important by frontline care professionals, they are less apt to study frontline care professionals’ actual health-promoting behavior. This study has provided insight into how health conceptions of frontline care professionals can be conceptualized in three dimensions. Future studies could complement this effort by conducting participant observations and experiments to study professionals’ behaviors more explicitly. Another limitation is that our strategy of snowball sampling does not guarantee representativeness (Johnson, 2014). The reason for this is that our initial respondents may have nominated professionals they know well or thought would be interested in the topic of health conceptions. However, this sampling strategy was necessary to recruit respondents matching our specific study focus, namely, professionals who work with clients with combined psychosocial problems and who are to some extent working together to care for clients. The goal of this research was to conceptualize health conceptions by exploring the health conception dimensions of various frontline professionals in social welfare and healthcare. We aimed for maximal variation in order to form conceptualizations; it was not our goal to make generalizable statements about different professional groups. The study's methodological approach allows for theoretical but not empirical generalization across contexts (Feldman and Orlikowski, 2011). It is possible that frontline care professionals who are not working with this specific client group perceive health differently. It is nonetheless likely that the findings are relevant for other areas of frontline care work distinguished by high levels of complexity and prolonged encounters between professionals and clients. These health conceptions may go beyond professionals working with this specific client group, especially because our respondents are often more general care professionals who also work with clients without combined problems. Comparative research is needed to further develop and validate this conceptualization of health by frontline care professionals. To this end, future research could compare the perception and use of health conception dimensions within different care organizations and between different types of frontline care professions to advance the theory of this field of study. It was also beyond the scope of this study to evaluate whether and how the use of different health conception dimensions results in better or worse outcomes for clients (Møller, 2022). These are important questions for future research.

## 8 Conclusions

Drawing on qualitative in-depth interviews among frontline professionals in healthcare and social welfare working with clients with psychosocial problems, this research found that professional health conceptions can be conceptualized along three dimensions that go beyond health definitions such as those studied by others (Huber et al., 2016). First, frontline care professionals define health in different ways. Second, they aim to interact on the same level as clients to make the relationship equal. Third, professionals say to place clients in their broader contexts in order to understand what kind of health goals and care are appropriate.

This study also explored the interplay between the different health conception dimensions and has shown that frontline care professionals combine health conception dimensions and health definitions when treating clients with combined problems.

## Funding

No specific funding was received for this research project.

## Data availability

The data underlying this article contains personally identifiable information regarding sensitive topics and vulnerable participants, and cannot be shared publicly due to the privacy of individuals who participated in the study. Disclosure of the data would violate the EU general Data Protection Regulation.

## Declaration of Competing Interest

The authors declare that they have no known competing financial interests or personal relationships that could have appeared to influence the work reported in this paper.

## Figures and Tables

**Table:** Respondent characteristics.

Respondent ID	Job position	Professional discipline
R1	Out-patient attendant	Social
R2	General practitioner	Medical
R3	Community police officer	Social
R4	Social case manager municipality	Social
R5	Client supporter	Social
R6	Out-patient attendant	Social
R7	Practice nurse mental healthcare	Medical
R8	General practitioner	Medical
R9	Recovery coach mental health	Mental
R10	Recovery coach mental health	Mental
R11	Mental health worker with police	Mental
R12	Manager multidisciplinary approach at municipality	Social
R13	Social psychiatric case manager	Social
R14	Community sports coach	Social
R15	Community sports coach (and dietician)	Social
R16	General practitioner	Medical
R17	Case manager social support law municipality	Social
R18	Prevention officer mental health	Mental
R19	Mental health worker	Mental
R20	Community sports coach	Social
R21	Community gardener	Social
R22	Police officer, specialist on people suffering confusion	Social
R23	Social psychiatric nurse	Mental

**Table:** 

Overarching topics	Goal	Topics interview questions & probes
Before the interview	Explaining nature and aim of research, reassuring confidentiality, handling of data, and seeking permission and consent	• Interview guide. Explaining aim of research project • Explaining handling of data • Asking for permission to audio record interview • Asking for informed consent
Introduction	Acquiring contextual knowledge Setting the scene of the interview and role expectations	• Professional background and experience • Current job and daily activities
Health views	Acquiring knowledge about health views	• Kinds of health views held • What is done to achieve this • Why is this important • Other desired outcomes • Changes over time
Health views in collaboration with other professionals	Acquiring knowledge about working with other professionals	• Kinds of professionals and organizations they work with • How forms of collaboration are organized • How the types of collaboration mentioned are experienced • How health views may align • Importance of collaboration for reaching health views
Health view in interaction with clients	Acquiring knowledge about working with clients	• Importance of client values and ideas • Situation in which client could be helped well > Why and how • Situation in which client could not be helped well > Why and how • How health views may align with clients > Weighing importance of client preferences • Experience of interaction with clients
Closing	Exploring other themes left unmentioned but of potential importance for the research Explaining overall planning of research project and thanking respondent for participation	• What are important values in their work? • Any other themes that respondents would like to address • Explaining further planning of the project

**Table:** Coding table of generic description of health conception dimensions.

	Mental healthcare Group=211	Medical healthcare Group=137	Social welfare Group=464	Totals
**Contextualization of other problems** **Gr**oup**=41**	8	12	24	44
**Contextualization of social context** **Gr**oup**=40**	9	8	20	37
**Defining competence Gr**oup**=66**	20	4	39	63
**Defining mental** **Gr**oup**=35**	10	6	18	34
**Defining physical Gr**oup**=10**	3	2	5	10
**Alignment_ being approachable** **Gr**oup**=13**	3	5	5	13
**Alignment seeking alignment** **Gr**oup**=62**	17	18	28	63
**Totals**	70	55	139	264

The number of quotes coded with health conception dimension per type of professional.

**Table:** Co-occurence coding table of health conception dimensions.

	Contextualization of other problems Group=41	Contextualizatoin of social context Group=40	Defining health competence Group=66	Defining health mental Group=35	Defining health Physical Group=10	Being approachable Group=13	Seeking alignment Group=62
**Contextualization of other problems Gr**oup**=41**	0	4	2	4	2	0	8
**Contextualization of social context Gr**oup**=40**	4	0	5	2	1	1	7
**Defining competence Gr**oup**=66**	2	5	0	9	2	0	4
**Defining health mental Gr**oup**=35**	4	2	9	0	2	0	4
**Defining health physical Gr**oup**=10**	2	1	2	2	0	0	2
**Being approachable Gr**oup**=13**	0	1	0	0	0	0	2
**Seeking alignment Gr**oup**=62**	8	7	4	4	2	2	0

Co-occurrence is established when respondents mention two dimensions in the same story within an interview.

## Appendix E Supplementary materials

Image, application 1

## Footnotes

1 Health conceptions refer to the perceptions of the professionals themselves, and the conceptualization is the result of our analysis.

2 Freely translated from Dutch : ‘Lullen tot je een ons weegt’ (respondent 3, police officer).
