# Supplementary material for: Professionals’ health conceptions of clients with psychosocial problems: An analysis based on an empirical exploration of semi-structured interviews
Source: Int J Nurs Stud Adv. 2023 Mar 5;5:100120. doi: 10.1016/j.ijnsa.2023.100120 (PMC11080445; doi:10.1016/j.ijnsa.2023.100120)
Supplement: Supplementary file 1 [file mmc1.docx]

**Consolidated criteria for reporting qualitative studies (COREQ): 32-item checklist for manuscript uploaded by Fia van Heteren.**

Domain 1: Research team and reflexivity

*Personal Characteristics*

1. Interviewer/facilitator

Which author/s conducted the interview or focus group?

The first author conducted the interviews.

1. Credentials

What were the researcher’s credentials? E.g. PhD, MD

The researcher and first author’s credentials are a Master of Arts in Anthropology of policy in practice and a Master of Science in Public Management.

1. Occupation

What was their occupation at the time of the study?

Their occupation at the time of the study was a PhD candidate at Leiden University Medical Centre and Leiden University.

1. Gender

Was the researcher male or female?

The researcher is female.

1. Experience and training

What experience or training did the researcher have?

During their Masters the researcher was trained in qualitative interviewing and in various types of interpretive study designs. Moreover, during their PhD trajectory they followed several courses in qualitative interviewing, ethnographic fieldwork and qualitative data analysis.

*Relationship with participants*

1. Relationship established

Was a relationship established prior to study commencement?

The researcher had not prior relationship with the research respondents.

7. Participant knowledge of the interviewer

What did the participants know about the researcher? e.g. personal goals, reasons for doing the

Research.

Before and during the interviews the researcher was open about their professional identity, personal goals and reasons for doing the research. They were briefed extensively and given enough time beforehand to decide on participation in the research.

1. Interviewer characteristics

What characteristics were reported about the interviewer/facilitator? e.g. Bias, assumptions,

reasons and interests in the research topic.

During the interviews, notes were made about possible biases, assumptions the researcher had about their respondents. Moreover, reflections were made about the interview experiences by the researcher. Also, all participants were asked about their experiences in participating in the research (see interview guide in manuscript). These reflections were all collected and reflected on again later during the analysis and writing up of the research. When the researcher’s position was expected to influence the results, details about these reflections would be shared in the manuscript.

Domain 2: study design

*Theoretical framework*

9. Methodological orientation and Theory

What methodological orientation was stated to underpin the study? e.g. grounded theory,

discourse analysis, ethnography, phenomenology, content analysis.

An interpretive research design was used and the study follows an inductive logic.

*Participant selection*

10. Sampling

How were participants selected? e.g. purposive, convenience, consecutive, snowball.

After a purposive introduction to the research field, snowball sampling was used to recruit respondents with specific criteria in mind.

11. Method of approach

How were participants approached? e.g. face-to-face, telephone, mail, email.

Email and face-to-face methods were used to approach participants.

12. Sample size

How many participants were in the study?

The study knows 23 participants.

13. Non-participation

How many people refused to participate or dropped out? Reasons?

One person refused to participate in the study because of lack of time.

Setting

14. Setting of data collection

Where was the data collected? e.g. home, clinic, workplace.

The interviews took place in a work setting chosen by the participant. Mostly in their offices or through online videocalls during Covid-19.

15. Presence of non-participants

Was anyone else present besides the participants and researchers?

No one else was present during the interviews.

16. Description of sample What are the important characteristics of the sample? e.g. demographic data, date.

Respondents were selected on theoretical grounds. All 23 respondents are frontline professionals in social welfare or (mental) healthcare working with clients with psychosocial problems in The Hague. Because this exploratory study aimed to gather a multitude of perspectives, the sample consisted of various kinds of frontline professionals, such as: ambulatory attendants, psychiatric nurses, community police officers and general practitioners. All respondents have been doing frontline work for years (Further respondent characteristics in appendix A).

*Data collection*

17. Interview guide

Were questions, prompts, guides provided by the authors? Was it pilot tested?

An interview guide was provided by the author (see appendix B in manuscript)

18. Repeat interviews

Were repeat interviews carried out? If yes, how many?

No repeat questions were carried out.

19. Audio/visual recording

Did the research use audio or visual recording to collect the data?

The researcher audio recorded all interviews.

20. Field notes

Were field notes made during and/or after the interview or focus group?

Jottings were made during the interviews and these were written out more extensively right after the interviews.

21. Duration

What was the duration of the interviews or focus group?

The duration of the interviews was between 45 and 90 minutes.

22. Data saturation

Was data saturation discussed?

Data saturation was reached when no new themes came up in the interviews.

23. Transcripts returned

Were transcripts returned to participants for comment and/or correction?

When desired, transcript were returned to participants for comments and/or correction. No respondents made use of the option to correct or comment on the transcript afterwards.

Domain 3: analysis and findings

*Data analysis*

24. Number of data coders How many data coders coded the data?

The first author was the main coder who coded all of the data. The second author was involved in checking the interpretation of the findings, including the meaning that has been given to the themes found. The third and fourth author were involved in further interpretation of these themes.

25. Description of the coding tree

Did authors provide a description of the coding tree?

Table 1 in the manuscript offers a description of the different main- and subthemes that were found during the coding process.

26. Derivation of themes

Were themes identified in advance or derived from the data?

Sensitizing concepts that guided our analysis were identified in the theoretical framework in advance, but final themes were derived inductively from the data.

27. Software

What software, if applicable, was used to manage the data?

The software package Atlas.ti version 7 was used to manage the data.

28. Participant checking

Did participants provide feedback on the findings?

Participants were not given the opportunity to provide feedback on the findings yet. However, we used data triangulation (after the semi-structured interviews we continued our research by doing participant observation in the same field) to make sure that our findings are credible.

*Reporting*

29. Quotations presented

Were participant quotations presented to illustrate the themes / findings? Was each

quotation identified? e.g. participant number.

Participant quotations were presented to illustrate the findings and each quotation was identified with a respondent number.

30. Data and findings consistent

Was there consistency between the data presented and the findings?

There was consistency between the data presented and the findings.

31. Clarity of major themes

Were major themes clearly presented in the findings?

Major theses were clearly presented in the findings section.

32. Clarity of minor themes

Is there a description of diverse cases or discussion of minor themes?

Apart from major themes, minor themes and diverse cases were given attention in the conclusion and discussion section.
